# Supplementary material for: Dual Ribosome Profiling reveals metabolic limitations of cancer and stromal cells in the tumor microenvironment
Source: Nat Commun. 2025 May 19;16:4652. doi: 10.1038/s41467-025-59986-7 (PMC12089342; doi:10.1038/s41467-025-59986-7)
Supplement: Supplementary file 1 — Supplementary information [file 41467_2025_59986_MOESM1_ESM.pdf]

Supplementary information

Dual Ribosome Profiling reveals metabolic limitations of cancer and stromal cells in the tumor microenvironment

Daniela Aviles-Huerta[1,2#], Del Pizzo Rossella[1,2#], Alexander Kowar[1,2], Ali Hyder Baig[1], Giuliana Palazzo[1,2], Ekaterina Stepanova[1], Cinthia Claudia Amaya Ramirez[1], Sara D'Agostino[1], Edoardo Ratto[3,2], Catarina Pechincha[3,2], Nora Siefert[3,2], Helena Engel[4,2], Shangce Du[4,5], Silvia Cadenas-De Miguel[6], Beiping Miao[4], Victor M. Cruz-Vilchez[1], Karin Müller-Decker[7], Ilaria Elia[6], Chong Sun[4], Wilhelm Palm[3], Fabricio Loayza-Puch[1,*]

#Equal contribution, *Correspondence to f.loayza-puch@dkfz-heidelberg.de

1 Translational Control and Metabolism, German Cancer Research Center (DKFZ), Heidelberg, Germany, 69120 Heidelberg, Germany.
2 Faculty of Biosciences, University of Heidelberg, Heidelberg, Germany
3 Division of Cell Signaling and Metabolism, German Cancer Research Center (DKFZ), Im Neuenheimer Feld 280, Heidelberg, Germany.
4 Immune Regulation in Cancer, German Cancer Research Center (DKFZ), Im Neuenheimer Feld 280, Heidelberg, Germany.
5 Faculty of Medicine, University of Heidelberg, Heidelberg. Germany
6 Department of Cellular and Molecular Medicine, KU Leuven, 3000 Leuven, Belgium
7 Core Facility Tumor Models, German Cancer Research Center (DKFZ), Im Neuenheimer Feld 280, Heidelberg, Germany

Supplementary Figures 1 to 8

Supplementary Figure 1.

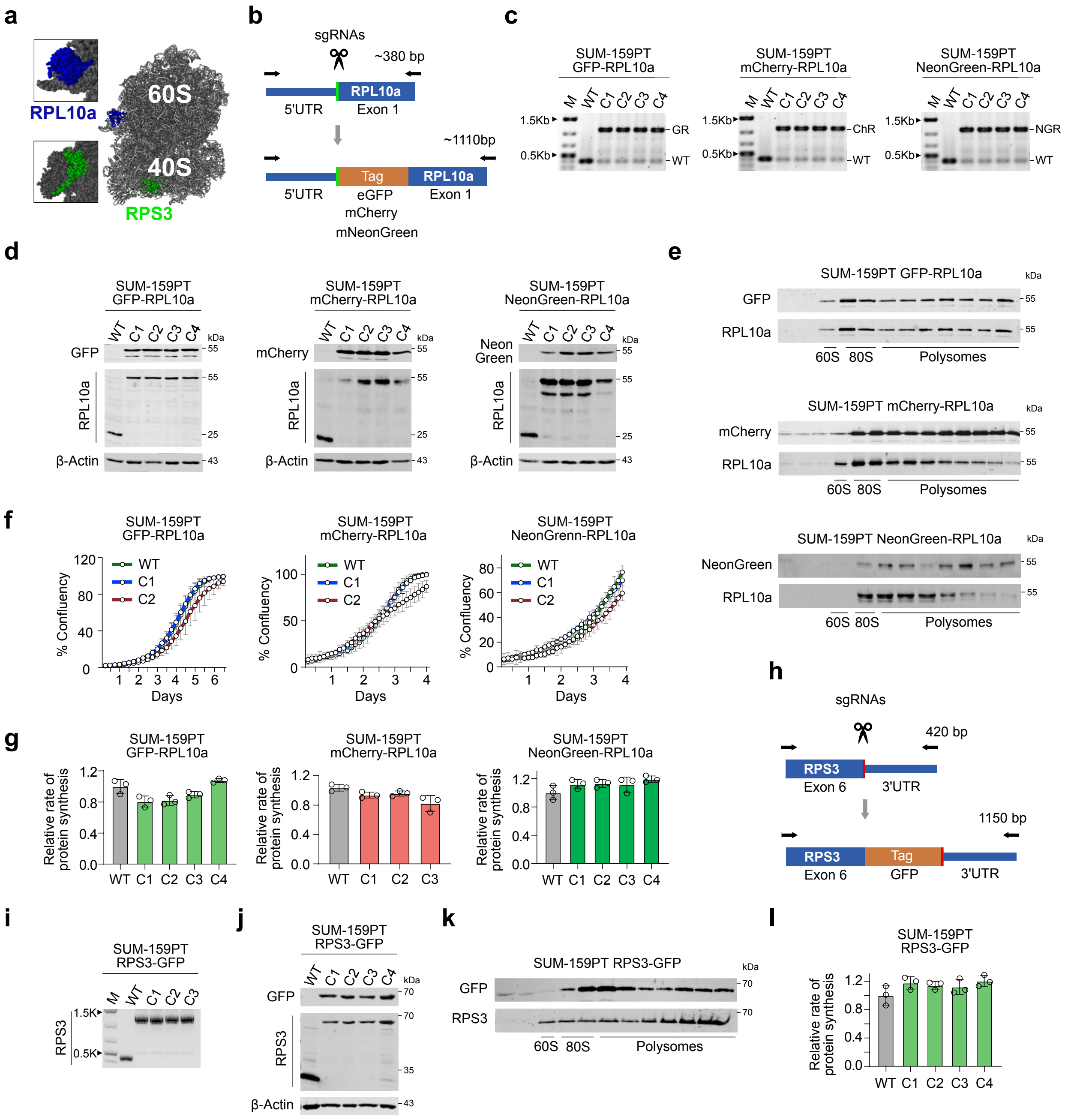

Supplementary Figure 1. Characterization of cell lines carrying tagged ribosomes.

a, The fluorescent tags indicated were endogenously incorporated at the N-terminus of RPL10a or the C-terminus of RPS3. The figure shows the location of both proteins in the large and small subunit of the ribosome, respectively.

b, Paired CRISPR–Cas9 nickase approach for tagging the *RPL10a* gene.

c, Gene editing was performed on SUM-159PT cells using CRISPR-Cas9 to tag either GFP-RPL10a, mCherry-RPL10a, or mNeonGreen-RPL10a to the N-terminus of RPL10a. Homozygous incorporation was confirmed through genotyping (GR, GFP-RPL10a; ChR, mCherry-RPL10a; NGR, mNeonGreen-RPL10a; WT, wild type).

d, Immunoblotting was performed on lysates from clones of the indicated cells, comparing WT with tagged-RPL10a.

e, Western blot analysis was performed using proteins isolated from individual fractions across the sucrose gradients of cell lysates from SUM-159PT cells expressing GFP-RPL10a, mCherry-RPL10a, or NeonGreen-RPL10a.

f, IncuCyte cell proliferation curves of SUM-159PT clones expressing GFP-RPL10a, mCherry-RPL10a, or NeonGreen-RPL10a. Data represent mean ± SD (n=7).

g, Protein synthesis rates were determined based on O-propargyl-puromycin (OP-Puro) incorporation in SUM-159PT clones expressing GFP-RPL10a, mCherry-RPL10a, or NeonGreen-RPL10a. Data represent mean ± SD from biologically independent experiments (n=3).

h, GFP was endogenously tagged at the C-terminus of RPS3 using a paired CRISPR–Cas9 nickase approach.

i, Genotyping of the homozygous incorporation of GFP in the C terminus of RPS3.

j, Western blot analysis of SUM-159PT WT or RPS3-GFP cell lysates.

k, Polysomal distribution of RPS3-GFP examined by Western blot analysis.

l, OP-Puro incorporation assay in SUM-159PT clones expressing RPS3-GFP. Data represent mean ± SD from biologically independent experiments (n=3).

Supplementary Figure 2.

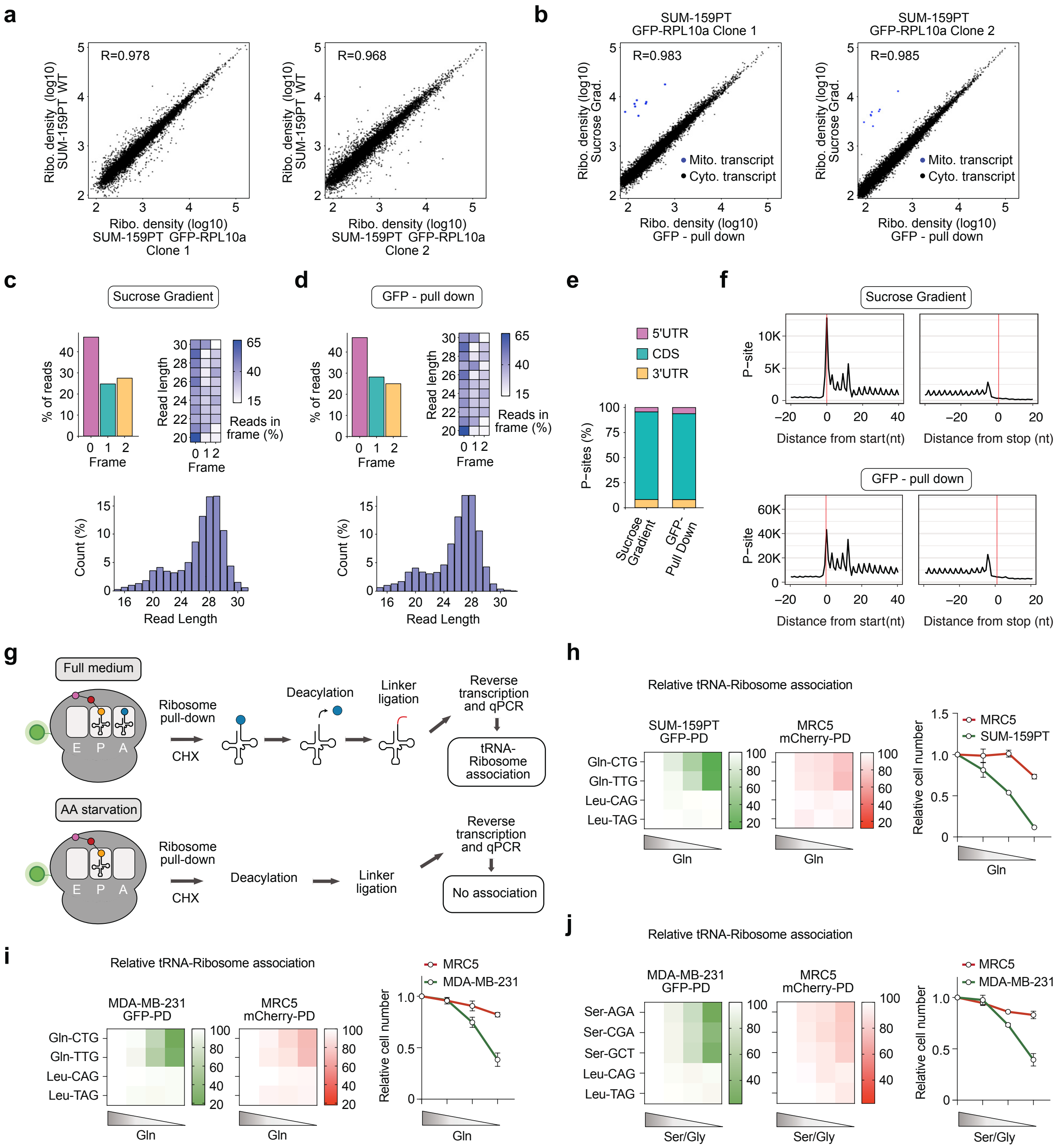

Supplementary Figure 2. Ribosome profiling and tRNA association studies in tagged breast cancer cell lines.

a, Scatter plot comparing RPF abundance between ribosome profiling datasets from SUM-159PT and SUM-159PT GFP-RPL10a clones. The Pearson correlation coefficient (R) is marked at the upper left corner. All libraries were prepared with conventional sucrose gradients.

b, Scatter plot comparing RPF abundance between ribosome profiling datasets generated from conventional sucrose gradients and GFP pull-downs from SUM-159PT-GFP-RPL10a clones. The Pearson correlation coefficient (R) is marked at the upper left corner. mtDNA-encoded transcripts (Mito. transcript) are underrepresented in the GFP pull-down libraries.

c-d, Frame and read-length distributions of the 5' end of RPFs from sucrose gradients and GFP pull-down libraries.

e, Percentage of reads mapping to the 5′ UTR, coding sequence (CDS), and 3′ UTR of mRNAs from datasets generated from conventional sucrose gradients or GFP pull-downs from SUM-159PT-GFP-RPL10a clones.

f, RPF Coverage around START and STOP codons in ribosome profiling libraries derived from sucrose gradients and GFP pull-downs.

g, Schematic of the tRNA-Ribosome association approach. CHX, Cycloheximide.

h-j, Ribosome–tRNA association assays in SUM-159PT-GFP-RPL10a (h), MDA-MB-231-GFP-RPL10a (i–j) and MRC5-mCherry-RPL10a cells. Cells were starved of either glutamine (glutamine concentrations: 4 mM, 2 mM, 1 mM, 0.5 mM, 0 mM; shading from light to dark) or serine/glycine (serine + glycine concentrations: 0.4 mM, 0.2 mM, 0.1 mM, 0.05 mM, 0 mM; shading from light to dark). Right panels show normalized cell numbers for each cell line at the indicated amino-acid concentrations. Cell number at each concentration is expressed relative to the untreated control measured 48 h after plating. Data are presented as mean ± SD (*n*=3). PD, pull-down.

Supplementary Figure 3.

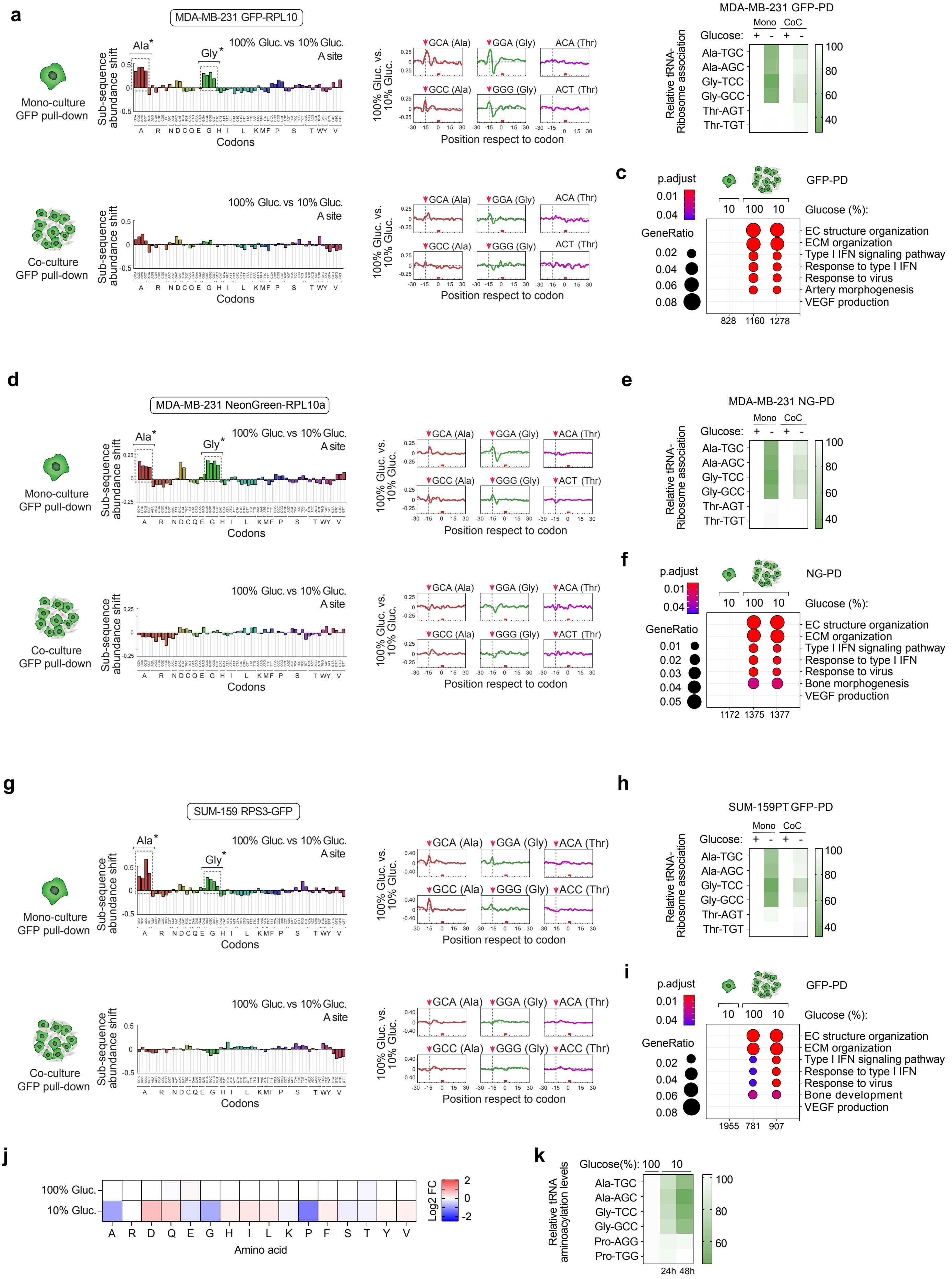

Supplementary Figure 3. DualRP measurements are independent of cell line, protein tag, or ribosomal subunit tagging.

a, d, g, Diricore analysis in three independent cell models. In each panel, the upper plots show mono-cultures of tagged cancer cells (a: MDA-MB-231-GFP-RPL10a; d: MDA-MB-231-NeonGreen-RPL10a; g: SUM-159PT-RPS3-GFP), and the lower plots show co-cultures with MRC5-mCherry-RPL10a fibroblasts. Cells were grown for 48 h in either full (100%, 25 mM glucose) or glucose-deprived (10%, 2.5 mM glucose) medium. *Out-of-frame analysis $P < 0.01$ for the GCA, GCC, GCG, GCT Ala codons and for the GGA, GGC, GGG Gly codons.

b, e, h, Ribosome–tRNA association was measured by immunoprecipitating tagged ribosomes from mono- and co-cultures of three cancer cell models with MRC5 fibroblasts. Cells were grown for 48 h in either full (+; 25 mM glucose) or glucose-deprived (–; 2.5 mM glucose) medium. (b) MDA-MB-231–GFP-RPL10a mono- and co-cultures. (e) MDA-MB-231–NeonGreen-RPL10a mono- and co-cultures. (h) SUM-159PT–RPS3-GFP mono- and co-cultures. Data represent mean ± SD (n=3). PD, pull-down.

c, f, i, Gene ontology enrichment of upregulated genes upon cancer–fibroblast interaction under glucose replete or deprived conditions. Ribo-Seq was performed on pull-downs (PD) from mono- and co-cultures of three cancer cell models with MRC5-mCherry-RPL10a fibroblasts grown for 48 h in full (+; 25 mM glucose) or glucose-deprived (–; 2.5 mM glucose) medium. Differential expression analysis identified genes upregulated during heterotypic interaction in: (c) MDA-MB-231-GFP-RPL10a cells, (f) MDA-MB-231-NeonGreen-RPL10a cells (NG), and (i) SUM-159PT-RPS3-GFP cells. For each model, significantly enriched Gene Ontology (GO) terms (p<0.05) are plotted with their GeneRatio (number of upregulated genes in the set/total genes in that set).

j, Heatmap representing changes of amino acid levels measured by LC-MS in SUM-159PT cells growing in full medium (100% Gluc., 25mM) or in limiting concentrations of glucose (10% Gluc., 2,5mM) for 48 hours. Values are shown as log2 (fold change). Data represent mean (n=3).

k, Aminoacylation assay in SUM-159PT breast cancer cells growing in full medium (100% Gluc., 25mM) or in limiting concentrations of glucose (10% Gluc., 2,5mM) for 24 and 48 hours. Data represent mean (n=3).

Supplementary Figure 4

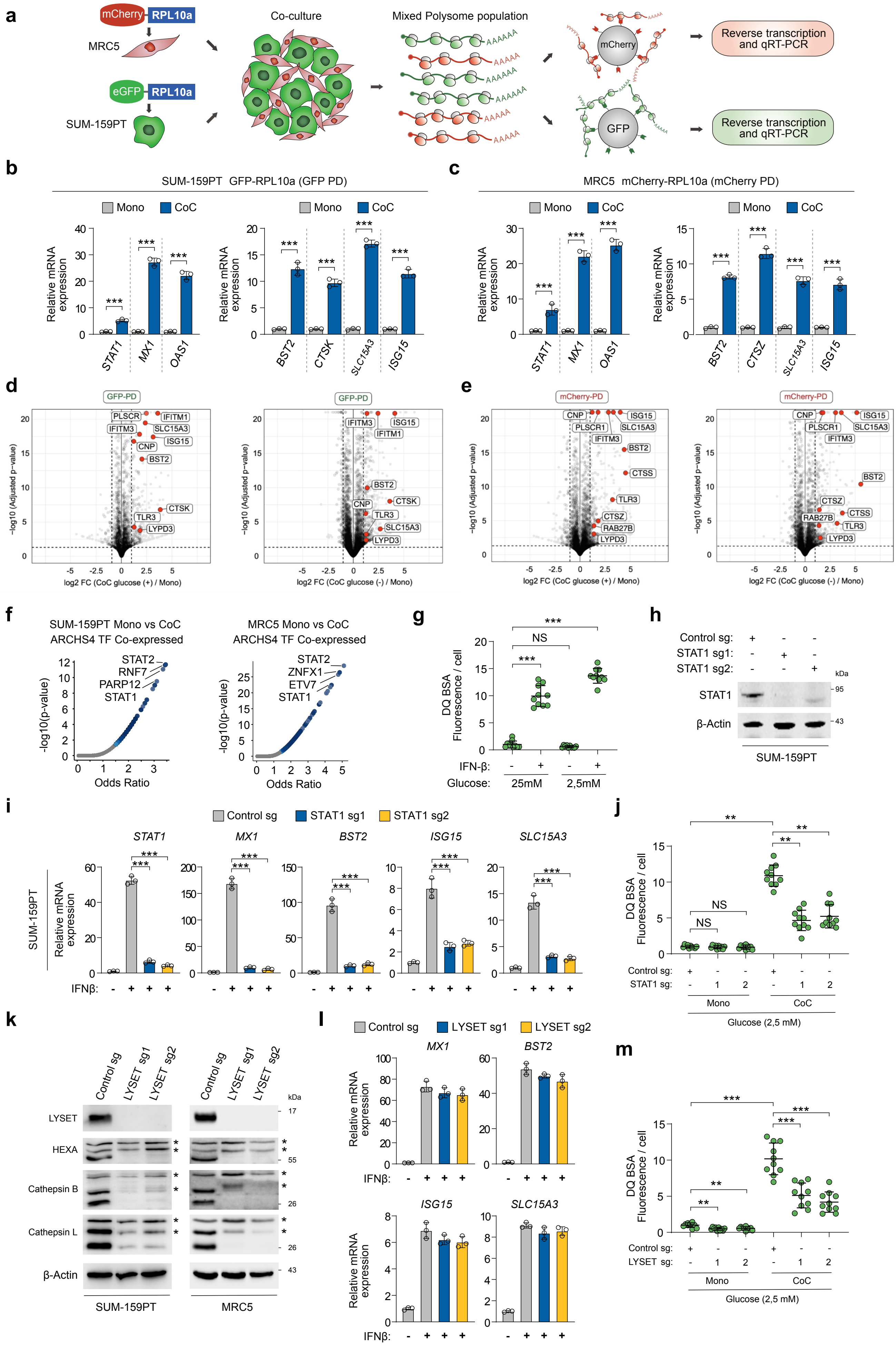

Supplementary Figure 4. Type I IFN signaling increases lysosomal catabolism upon heterotypic cell interactions.

a, Schematic diagram illustrating the isolation and quantification of polysome-associated transcripts from co-cultures of SUM-159PT-GFP-RPL10a breast cancer cells and MRC5-mCherry-RPL10a fibroblasts.

b–c, SUM-159PT-GFP-RPL10a cancer cells and MRC5-mCherry-RPL10a fibroblasts were grown either as monocultures or as 1:1 co-cultures for 48 h. RNA was extracted from pull-down (PD) samples and analyzed by qRT-PCR for the indicated genes in (b) cancer cells and (c) fibroblasts. Data represent mean ± SD from biologically independent experiments (n=3); p-values were calculated using a two-tailed unpaired t-test. ***p<0.001. PD, pull-down. Source data including exact p-values are provided as Source Data file.

d-e, Volcano plot illustrating differential expression of genes based on Ribo-seq counts in mono- and co-cultures of SUM-159PT GFP-RPL10a (d) and MRC5 mCherry-RPL10a (e) grown in full ((+), 25mM) or glucose-deprived ((-), 2,5mM) medium. Selected lysosomal genes are highlighted in red. PD, pull-down.

f, Volcano plot illustrating the significance of the upregulated lysosomal gene set in relation to its odds ratio. Every data point represents an individual gene set. The x-axis, represents the odds ratio calculated for each gene set, while the y-axis shows the negative logarithm of the p-value associated with the gene set. Larger data points displayed in blue indicate statistically significant terms (p-value < 0.05), while smaller gray data points represent terms that did not reach statistical significance. The intensity of the blue color of a data point corresponds to its level of significance, with darker blue points indicating higher significance.

g, DQ BSA quantification in SUM-159PT cells treated with vehicle or IFN-β (10 ng/mL) for 48hrs. Cells were grown in full medium (Glucose 25mM) or in low-glucose (2,5mM) medium. Data represent mean ± SD from biologically independent experiments (n=10); p-values were calculated using a two-tailed unpaired t-test. NS, non-significant; ***p<0.001. Source data including exact p-values are provided as Source Data file.

h, Western blots on cell extracts from SUM-159PT-GFP-RPL10a cells transduced with sgRNAs targeting STAT1.

i, qRT-PCR quantification of the indicated genes in SUM-159PT-GFP-RPL10a transduced with sgRNAs targeting STAT1 and treated with IFN-β (10 ng/mL) for 24hrs. Data represent mean ± SD from biologically independent experiments (n=3); p-values were calculated using a two-tailed unpaired t-test. ***P < 0.001. Source data including exact p-values are provided as Source Data file.

j, Quantification of DQ BSA fluorescence in SUM-159PT GFP-RPL10a transduced with sgRNAs targeting the STAT1 gene or a control sequence growing as mono- or co-cultures (CoC) with MRC5 cells. Cells were grown in low-glucose condition (2,5mM). Data represent

mean ± SD from biologically independent experiments (*n*=10); *p*-values were calculated using a two-tailed unpaired *t*-test. NS, non-significant; $^{**}P < 0.01$. Source data including exact *p*-values are provided as Source Data file.

k, Western blot on cell lysates from SUM-159PT-GFP-RPL10a (left panel) and MRC5-mCherry-RPL10a (right panel) cells transduced with sgRNAs targeting LYSET. *Denotes immature lysosomal enzymes.

l, qRT-PCR assay of the indicated genes in SUM-159PT GFP-RPL10a transduced with sgRNAs against LYSET and treated with IFNβ (10 ng/mL) for 24hrs. Data represent mean ± SD from biologically independent experiments (*n*=3).

m, Fluorescence quantification of DQ BSA in SUM-159PT GFP-RPL10a cells transduced with sgRNAs targeting either the LYSET gene or a control sequence, cultured as mono- or co-cultures (CoC) with MRC5 cells. The cells were maintained under low-glucose conditions (2.5 mM). Data are presented as mean ± SD from biologically independent experiments (*n*=10); *p*-values were calculated using a two-tailed unpaired *t*-test. $^{**}P < 0.01$; $^{***}P < 0.001$. Source data including exact *p*-values are provided as Source Data file.

Supplementary Figure 5.

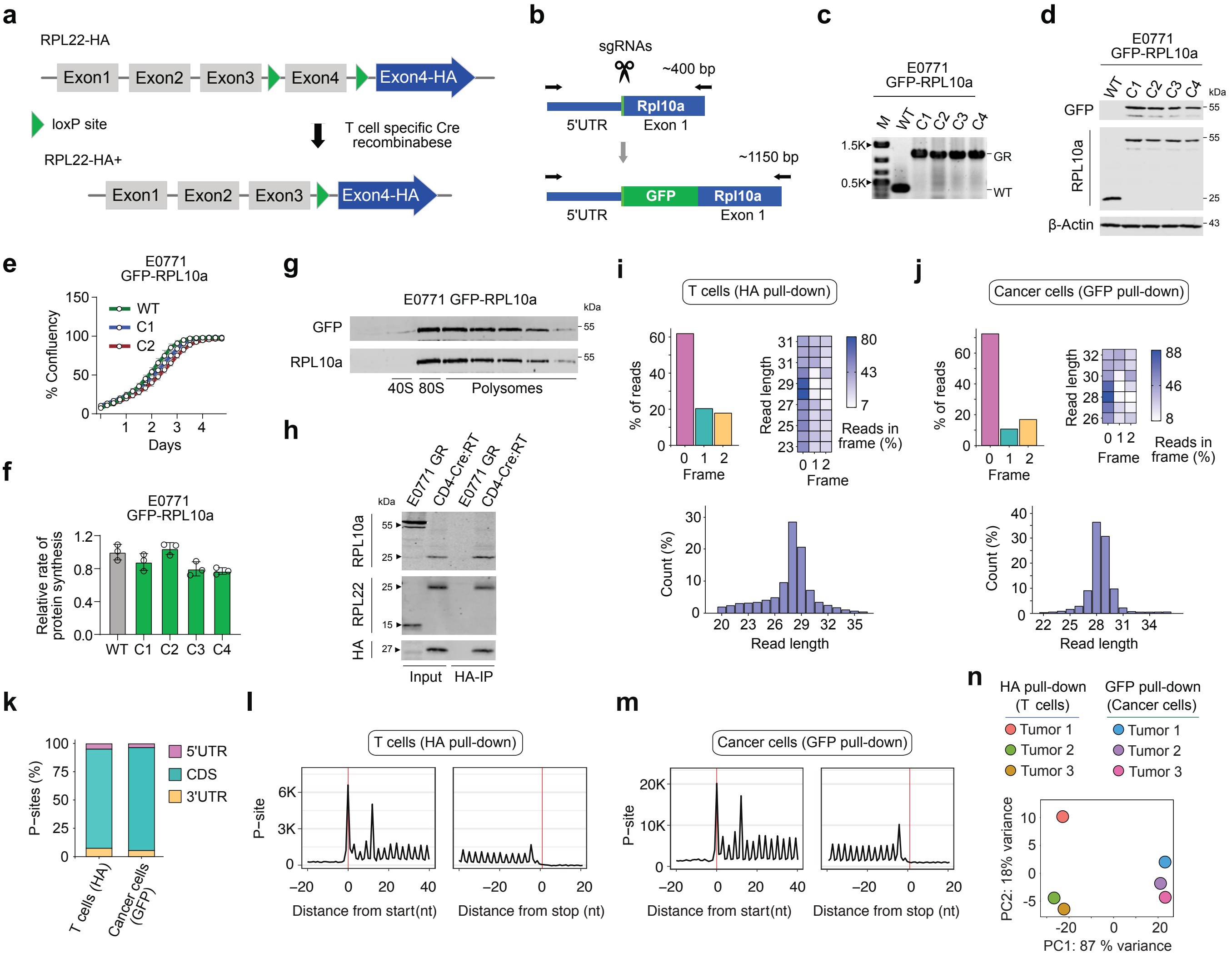

Supplementary Figure 5. DualRP enables the study of ribosome occupancy in multiple cell compartments of the TME.

a, Schematic diagram of CD4-Cre:RiboTag mice generation. The *Rpl22* gene was subjected to targeted modification through homologous recombination, where loxP sites were introduced to flank the final exon (Exon4). Downstream of the original Exon 4, a new Exon 4 was inserted into the *Rpl22* locus sequence. Activation of the CD4-Cre driver cleaved the loxP sites flanking the initial Exon 4, excising of the floxed exon. Consequently, the Exon4 containing the HA tag becomes incorporated into the RPL22 mRNA, giving rise to the production of RPL22 with an HA tag specifically in T cells (depicted in blue).

b, Paired CRISPR–Cas9 nickase approach for tagging the mouse *Rpl10a* gene.

c, Gene editing in E0771 cells to incorporate GFP to the N-terminus of RPL10a. Homozygous incorporation was confirmed by PCR.

d, Western blot on lysates from clones of E0771 GFP-RPL10a cells, comparing WT or tagged-RPL10a.

e, IncuCyte cell proliferation curves of E0771 clones expressing GFP-RPL10a. Data represent mean ± SD (*n*=6).

f, OP-Puro incorporation assay in E0771 clones expressing GFP-RPL10a. Data represent mean ± SD from biologically independent experiments (*n*=3).

g, Immunoblot assay was conducted using proteins isolated from individual fractions across the sucrose gradients of cell lysates from E0771 cells expressing GFP-RPL10a.

h, Spleens from CD4-Cre:RiboTag (CD4-Cre:RT) mice and E0771 GFP-RPL10a (E0771 GR) cells were lysed and then subjected to immunoprecipitation (IP) using an HA antibody. Both the input and IP fractions were subsequently probed by Western blotting with the specified antibodies.

i-j, Frame and read-length distributions of the 5' end of RPFs from ribosome profiling libraries prepared from anti-HA and anti-GFP pull-downs of E0771 tumors.

k, Percentage of reads mapping to the 5′ UTR, coding sequence (CDS), and 3′ UTR of mRNAs from data sets generated from anti-HA and anti-GFP pull-downs of E0771 tumors**.**

l-m, RPF Coverage around START and STOP codons in ribosome profiling libraries derived from the indicated tumor cell compartments.

n, Principal Component Analysis (PCA) from ribosome profiling libraries generated from the indicated pull-downs of E0771 tumors.

Supplementary Figure 6.

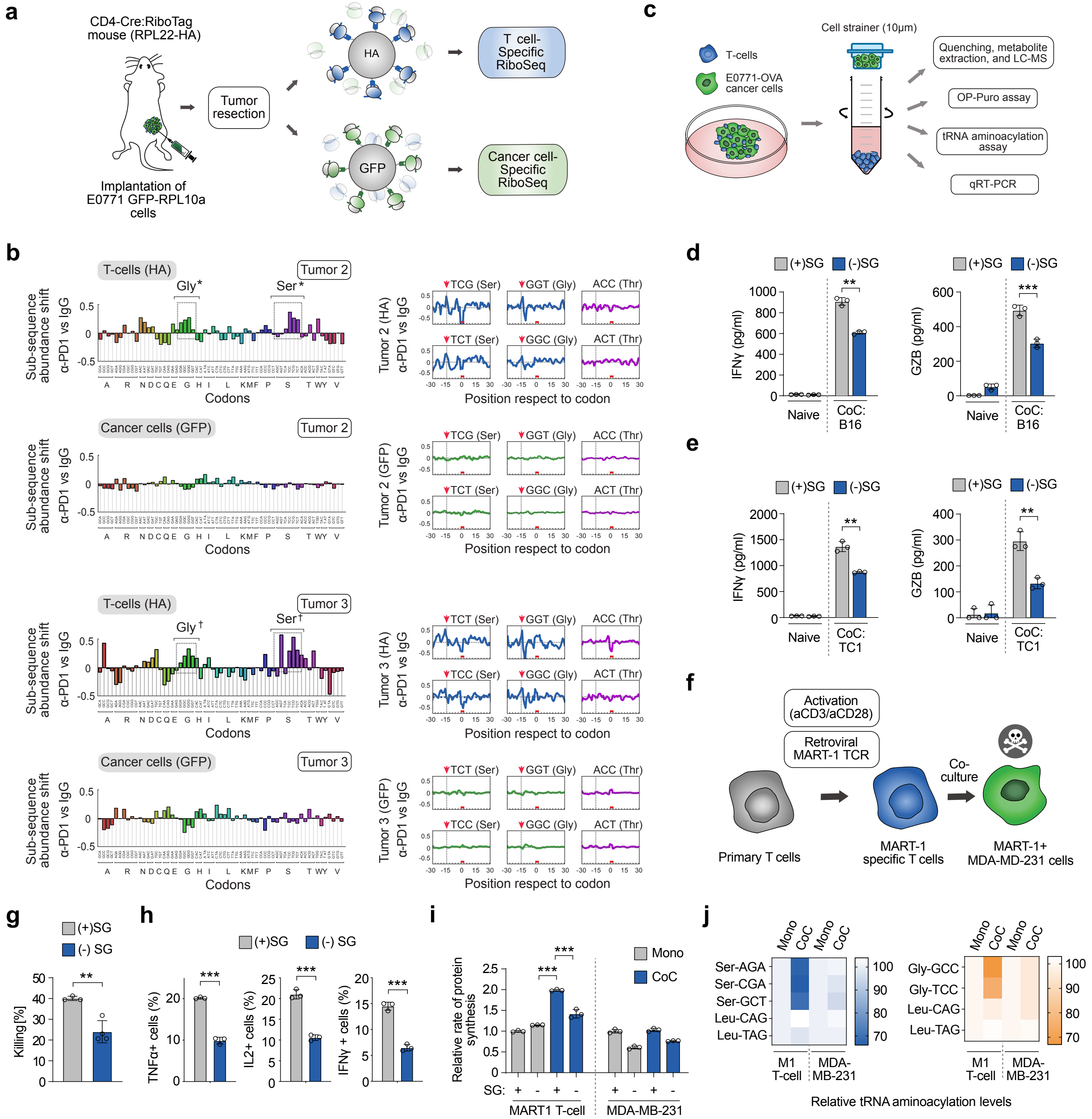

Supplementary Figure 6. DualRP identifies serine and glycine as crucial nutrients for T cell protein synthesis in the TME during immune checkpoint blockade.

a, Schematic depicting the *in vivo* Dual-RP experiment. Tumors are flash frozen after resection, lysed, and ribosomes from each cell type are immunoprecipitated using either anti-HA- or anti-GFP-coated beads.

b, Diricore analysis of independent tumors treated as described in Fig. 4a. Ribosome stalling in tumor-infiltrated T cells (HA) and E0771 tumor cells (GFP) are shown in the upper and lower panels, respectively. Ribosome density at the A site is shown. * Out-of-frame analysis for tumor 2, $P < 0.01$ for the TCC, TCG, TCT Ser codons and for the GGA, GGC, GGG Gly codons. † Out-of-frame analysis for tumor 3, $P < 0.01$ for the AGT, TCC, TCG, TCT Ser codons and for the GGC, GGG, GGT Gly codons.

c, Schematic of the cell-size-based separation of cancer cells and CD8+ T cells.

d-e, Quatification of IFNγ and GzmB levels by CD8+ T cells when co-culture with B16-OVA (d) or TC1-OVA (e) cells in full medium or serine/glycine-deprived medium. Data represent mean ± SD from biologically independent experiments (*n*=3); *p*-values were calculated using a two-tailed unpaired *t*-test. **$p < 0.01$; ***$p < 0.001$. Source data including exact *p*-values are provided as Source Data file.

f, Schematic representation of the human co-culture system of antigen-specific T cells and breast cancer cells. T cells were sourced from human peripheral blood mononuclear cells (PBMCs), then activated, and subsequently modified with a MART-1-specific T cell receptor (TCR). Following this, MDA-MD-231 cells expressing HLA-A2 were loaded with MART-1 peptide and co-cultured with the T cells carrying the engineered TCR.

g-h, Killing efficiency (g) and quantification of TNF, IL-2, and IFNγ levels produced by MART-1 T cells (h) when co-cultured with MDA-MD-231 in full medium or serine/glycine-deprived medium. Data represent mean ± SD from biologically independent experiments (*n*=3); *p*-values were calculated using a two-tailed unpaired *t*-test. ***P < 0.001. Source data including exact *p*-values are provided as Source Data file.

i, OP-Puro incorporation assay in MART-1 T cells and MDA-MB-231 breast cancer cells growing as mono- or co-cultures. Cell were grown in full medium (SG+) or serine/glycine-deprived medium (SG-). Data represent mean ± SD from biologically independent experiments (*n*=3); *p*-values were calculated using a two-tailed unpaired *t*-test. ***P < 0.001. Source data including exact *p*-values are provided as Source Data file.

j, Aminoacylation assay in MART-1 (M1) T cells and MDA-MB-231 breast cancer cells growing as mono- or co-cultures. Ser-tRNAs, Gly-tRNAs and control Leu-tRNAs were analyzed. Data represent mean ± SD (*n*=3).

Supplementary Figure 7

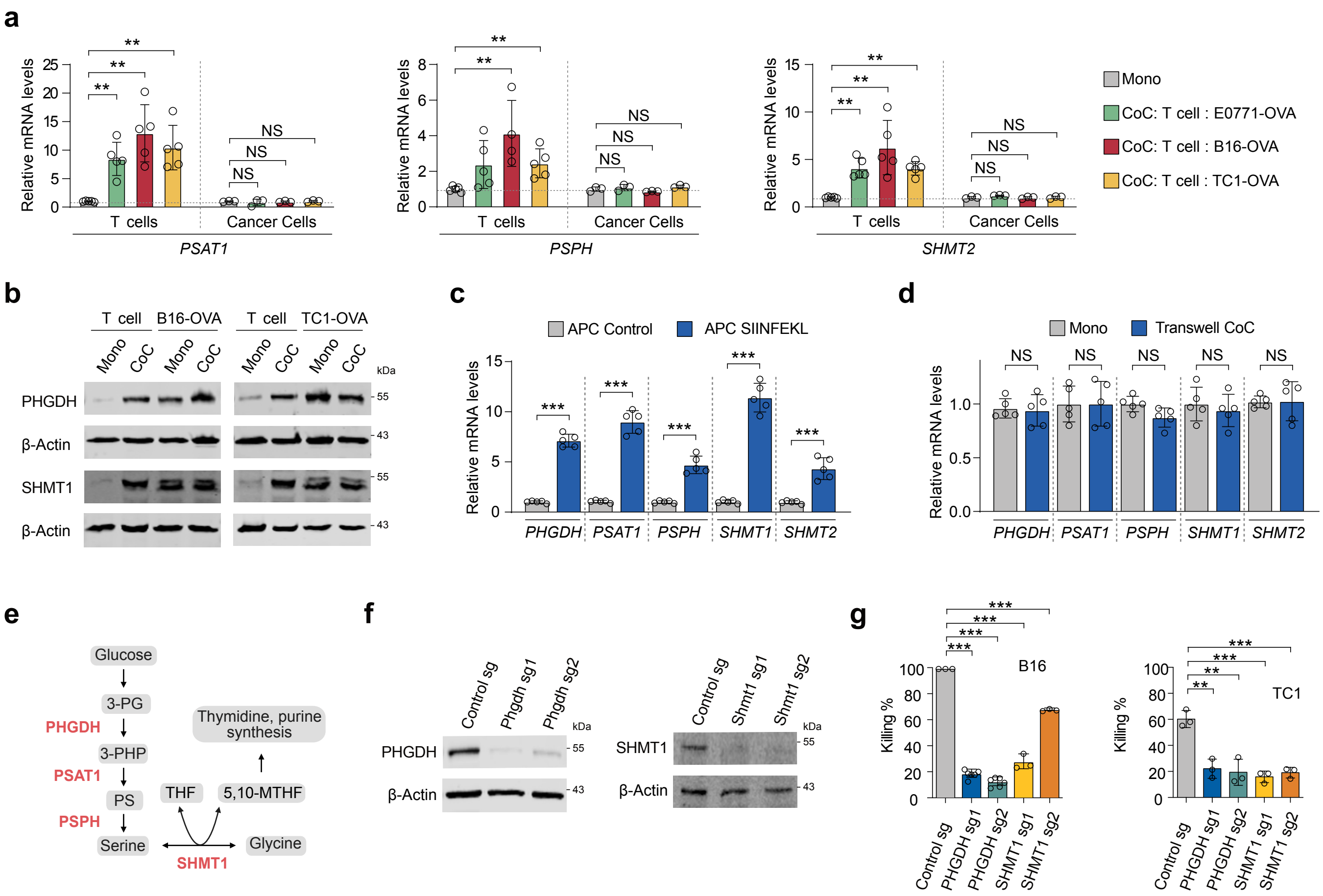

Supplementary Figure 7. The serine synthesis and one-carbon metabolism pathways are required for efficient cytotoxic activity in CD8$^+$ T cells.

a-b, qRT-PCR (i) and Western blot analysis (j) were carried out for the specified genes in co-cultures of OT-I cells and cancer cell lines expressing OVA. Cell separation based on size was performed after 24 hours of co-culture (refer to Figure S6c). Data represent mean ± SD from biologically independent experiments (*n*=5 for T cells and *n*=3 for cancer cells). *p*-values were calculated using a two-tailed unpaired *t*-test. NS, not significant; $^{**}p<0.01$. Source data including exact *p*-values are provided as Source Data file.

c, qRT-PCR of the indicated genes in OT-I T cells co-cultured with the antigen presenting cell (APC) line DC 2.4 treated with vehicle (APC control) or pulsed with SIINFEKL peptide (APC SIINFEKL). Data represent mean ± SD from biologically independent experiments (*n*=5). *p*-values were calculated using a two-tailed unpaired *t*-test. $^{***}p<0.001$. Source data including exact *p*-values are provided as Source Data file.

d, qRT-PCR quantification of the indicated genes in OT-I T cells co-cultured with E0771-OVA cells in a transwell plate to prevent direct physical contact. T cells were activated for 72h with anti-CD3, anti-CD28 and IL-2 before co-culture. Data represent mean ± SD from biologically independent experiments (*n*=5). *p*-values were calculated using a two-tailed unpaired *t*-test. NS, non-significant. Source data including exact *p*-values are provided as Source Data file.

e, Schematic representation of the serine synthesis and the one-carbon metabolism pathway. 3-PG, 3-phosphoglycerate; 3-PHP, 3-phosphohydroxypyruvate; PS, phosphoserine; PHGDH, 3-phosphoglycerate dehydrogenase; PSAT1, phosphoserine amino-transferase1; PSPH, phosphoserine phosphatase; THF, tetrahydrofolate; 5,10-MTHF, 5,10-methylene tetrahydrofolate; SHMT1, serine hydroxymethyltransferase.

f, Western blots depicting PHGDH (left panels) or SHMT1 (right panels) protein levels in mouse CD8+ T cells transduced with either control or sgRNAs targeting the *Phgdh* or *Shmt1* genes.

g, Cytotoxicity assay was conducted using CD8+ T cells expressing sgRNAs targeting *Phgdh* or *Shmt1*. These T cells were co-cultured with pulsed B16 (left panel) or TC1 cells (right panel) for a duration of 24 hours. Data represent mean ± SD from biologically independent experiments (*n*=3 or *n*=6). *p*-values were calculated using a two-tailed unpaired *t*-test. $^{**}p<0.01$; $^{***}p<0.001$. Source data including exact *p*-values are provided as Source Data file.

Supplementary Figure 8.

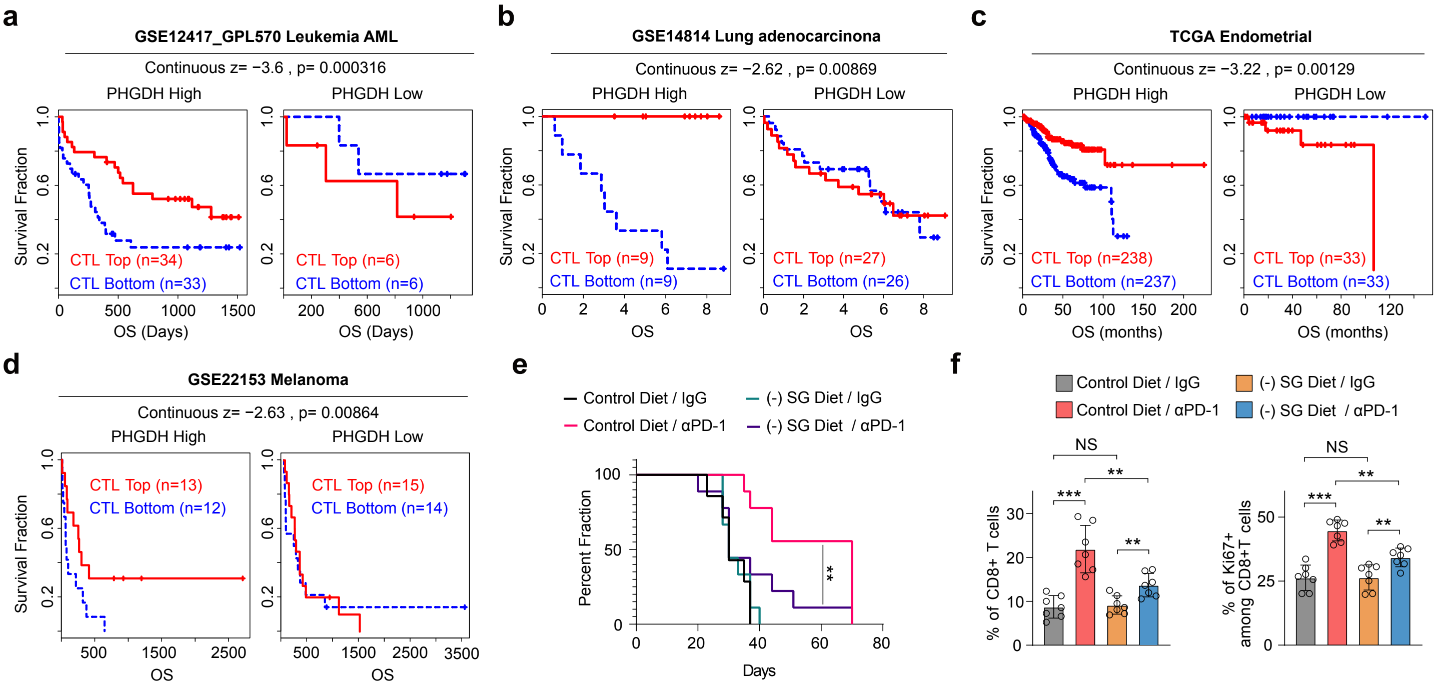

Supplementary Figure 8. PHGDH predicts cancer immunotherapy outcomes in patients.
a-d, TIDE analyses were conducted on PHGDH expression within T cell dysfunction signatures linked to increased survival in patients with Acute Myeloid Leukemia (AML) (a), Lung Adenocarcinoma (b), Endometrial Cancer (c), and Melanoma (d).
e, Survival over time was assessed in mice inoculated with E0771 tumor cells and fed either a control or a (-) SG diet 7 days later. When tumors became palpable, the animals were treated with either IgG (2 μg/μl) or anti-PD1 (2 μg/μl). ** $p<0.01$ by log-rank (Mantel–Cox) test.
f, Quantification of the proportion of CD8+ T cells in tumors. Data represent mean ± SD from biologically independent experiments ($n=7$). p-values were calculated using a two-tailed unpaired t-test. NS, not significant; ** $p<0.01$; *** $p<0.001$. Source data including exact p-values are provided as Source Data file.
