# Supplementary material for: Dual Ribosome Profiling reveals metabolic limitations of cancer and stromal cells in the tumor microenvironment
Source: Nat Commun. 2025 May 19;16:4652. doi: 10.1038/s41467-025-59986-7 (PMC12089342; doi:10.1038/s41467-025-59986-7)
Supplement: Supplementary file 3 — Reporting Summary [file 41467_2025_59986_MOESM3_ESM.pdf]

nature portfolio

Corresponding author(s): Fabricio Loayza-Puch

Last updated by author(s): Apr 23, 2025

Reporting Summary

Nature Portfolio wishes to improve the reproducibility of the work that we publish. This form provides structure for consistency and transparency in reporting. For further information on Nature Portfolio policies, see our Editorial Policies and the Editorial Policy Checklist.

Statistics

For all statistical analyses, confirm that the following items are present in the figure legend, table legend, main text, or Methods section.

n/a	Confirmed	
☐	☒	The exact sample size (*n*) for each experimental group/condition, given as a discrete number and unit of measurement
☒	☐	A statement on whether measurements were taken from distinct samples or whether the same sample was measured repeatedly
☐	☒	The statistical test(s) used AND whether they are one- or two-sided<br>*Only common tests should be described solely by name; describe more complex techniques in the Methods section.*
☐	☒	A description of all covariates tested
☒	☐	A description of any assumptions or corrections, such as tests of normality and adjustment for multiple comparisons
☐	☒	A full description of the statistical parameters including central tendency (e.g. means) or other basic estimates (e.g. regression coefficient) AND variation (e.g. standard deviation) or associated estimates of uncertainty (e.g. confidence intervals)
☐	☒	For null hypothesis testing, the test statistic (e.g. *F*, *t*, *r*) with confidence intervals, effect sizes, degrees of freedom and *P* value noted<br>*Give P values as exact values whenever suitable.*
☒	☐	For Bayesian analysis, information on the choice of priors and Markov chain Monte Carlo settings
☒	☐	For hierarchical and complex designs, identification of the appropriate level for tests and full reporting of outcomes
☐	☒	Estimates of effect sizes (e.g. Cohen's *d*, Pearson's *r*), indicating how they were calculated

Our web collection on statistics for biologists contains articles on many of the points above.

Software and code

Policy information about availability of computer code

Data collection	Flow cytometric analysis was performed using a FACSCanto Analyzer (BD Biosciences, New Jersey, USA).<br><br>Proliferation analyses were conducted using the IncuCyte S3 Live-Cell Analysis System (Sartorius, Göttingen, Germany).<br><br>Ribo-seq libraries were prepared as previously described (see Methods section) and sequenced as single-end, 100 bp reads on one lane of a NextSeq 2000 sequencer (Illumina).<br><br>qPCR data were acquired using the QuantStudio 3 Real-Time PCR System.<br><br>Flow cytometry data were analyzed using FACSDiva software, Cytek SpectroFlo, and FlowJo 10.4 (BD Biosciences).
Data analysis	Incucyte data was analyzed using the IncuCyte 2020C software<br><br>Differential gene expression analysis was performed with the R package DESeq2 v1.26.0<br><br>Gene set enrichment analyzes were performed utilizing the R package clusterProfiler v3.12.0

For manuscripts utilizing custom algorithms or software that are central to the research but not yet described in published literature, software must be made available to editors and reviewers. We strongly encourage code deposition in a community repository (e.g. GitHub). See the Nature Portfolio guidelines for submitting code & software for further information.

Data

Policy information about availability of data

All manuscripts must include a data availability statement. This statement should provide the following information, where applicable:

- Accession codes, unique identifiers, or web links for publicly available datasets
- A description of any restrictions on data availability
- For clinical datasets or third party data, please ensure that the statement adheres to our policy

The sequence data from this study have been submitted to NCBI BioProject (http://www.ncbi.nlm.nih.gov/bioproject) under BioProject number PRJNA1024949.

Research involving human participants, their data, or biological material

Policy information about studies with human participants or human data. See also policy information about sex, gender (identity/presentation), and sexual orientation and race, ethnicity and racism.

Reporting on sex and gender	N/A
Reporting on race, ethnicity, or other socially relevant groupings	N/A
Population characteristics	N/A
Recruitment	N/A
Ethics oversight	N/A

Note that full information on the approval of the study protocol must also be provided in the manuscript.

Field-specific reporting

Please select the one below that is the best fit for your research. If you are not sure, read the appropriate sections before making your selection.

☒ Life sciences ☐ Behavioural & social sciences ☐ Ecological, evolutionary & environmental sciences

For a reference copy of the document with all sections, see nature.com/documents/nr-reporting-summary-flat.pdf

Life sciences study design

All studies must disclose on these points even when the disclosure is negative.

Sample size	Sample sizes were determined based on prior knowledge of good sample sizes to ensure adequate data for reliable assessments as well as feasibility sequencing data generation. Sample sizes are indicated in the figure legends.
Data exclusions	Data was excluded based on QC results for individual assays.
Replication	All experiments were performed in at least three biological replicates and specific sample sizes are mentioned in the figure legends. Most experiments contain statistical analysis and significances of the results are indicated.
Randomization	Animal experiments were performed in randomized order.
Blinding	The investigators were not blinded to group allocation due to fixed experimental groups used for comparison.

Reporting for specific materials, systems and methods

We require information from authors about some types of materials, experimental systems and methods used in many studies. Here, indicate whether each material, system or method listed is relevant to your study. If you are not sure if a list item applies to your research, read the appropriate section before selecting a response.

Materials & experimental systems

n/a	Involved in the study	
☐	☒	Antibodies
☐	☒	Eukaryotic cell lines
☒	☐	Palaeontology and archaeology
☐	☒	Animals and other organisms
☒	☐	Clinical data
☒	☐	Dual use research of concern
☒	☐	Plants

Methods

n/a	Involved in the study	
☒	☐	ChIP-seq
☐	☒	Flow cytometry
☒	☐	MRI-based neuroimaging

Antibodies

Antibodies used

RPL10a (Abcam; cat. Ab174318, 1:1,000), RPS3 (Cell Signaling; cat. 9538, 1:1,000), eGFP (Santa Cruz; cat. sc-9996, 1:1,000), mCherry (Abcam; cat. Ab167453 1:1,000), Neongreen (Chromotek; cat. 32F6, 1:1,000), RPL22 (Santa Cruz; cat. sc-373993 1:500), HA-tag (Biolegend; cat. 901501, 1:1000), STAT1 (Cell Signaling; cat. 9172, 1:1,000), PHGDH (Sigma; cat. HPA021241, 1:1,000), SHMT1 (Cell Signaling;cat. 80715, 1:1,000), LYSET (Atlas Antibodies; cat. HPA048559, 1:1,000), Cathapsin B (R&D Systems; cat. AF953, 1:1000), Cathapsin L (R&D Systems; cat. AF952 1:1000), HEXA (R&D Systems; cat. AF6237, 1:1000).

Validation

Antibodies were purchased from the above stated companies. Antibodies are well described and published elsewhere. information can be sought from the manufactures website under the respective catalog number.

Eukaryotic cell lines

Policy information about cell lines and Sex and Gender in Research

Cell line source(s)

SUM-159PT (BioIVT, CVCL_5423), MDA-MD-231 (ATCC ,HTB-26), MRC5 (ATCC, CCL-171), E0771 (CH3 Bio Systems, 94001), TC1 (ATCC, CRL-2785), B16-F10 (ATCC CRL-6475)

Authentication

All cell lines were authenticated using the Multiplex cell line authentication test by Multiplexion (Heidelberg Germany)

Mycoplasma contamination

All cell lines were controlled for contamination using the LookOut Mycoplasma PCR Detection Kit (Sigma)

Commonly misidentified lines
(See ICLAC register)

No commonly misidentified cell lines were used.

Animals and other research organisms

Policy information about studies involving animals; ARRIVE guidelines recommended for reporting animal research, and Sex and Gender in Research

Laboratory animals

Mus musculus, C57BL/6, 6-8 weeks NSG, C57BL/6J (Strain Code 027), OT-1, Rpl22 x CD4-Cre and P14 x CD4-Cre x Rosa-LSL-Cas9-GFP

Wild animals

No wild animals were used in this study

Reporting on sex

Because this study focuses on female breast cancer, all animals used were female.

Field-collected samples

No field-collected samples were used in this study

Ethics oversight

Mice were housed under standard laboratory conditions. Experimental and control animals were co housed under the same conditions to minimize environmental variables. A 12-hour light/12-hour dark cycle was maintained, with the ambient temperature regulated at 22 ± 2°C and relative humidity set between 45% and 65%. Animals were provided with unrestricted access to food and water and were housed in groups of 3 to 5 per cage. All mice were kept in a pathogen-free environment and handled in accordance with protocols approved by the German Cancer Research Center and the supervisory authority (Regierungspräsidium Karlsruhe; G57-20), in compliance with the German Animal Protection Law and the European Directive 2010/63/EU on the protection of animals used for scientific purposes.

Animals were euthanized by cervical dislocation when any of the following humane endpoints were met: tumor volume ≥ 1.5 cm^3 or ≥ 1.5 cm in one dimension (tumor size was determined by caliper measurement), tumor ulceration, > 20% weight loss relative to age?, sex?, strain?, and diet?matched controls, or signs of distress (apathy, reduced food/water intake, respiratory difficulty, or abnormal posture/movement). Animals were monitored every 2-4 days.

Note that full information on the approval of the study protocol must also be provided in the manuscript.

Plants

Seed stocks

Report on the source of all seed stocks or other plant material used. If applicable, state the seed stock centre and catalogue number. If plant specimens were collected from the field, describe the collection location, date and sampling procedures.

Novel plant genotypes

Describe the methods by which all novel plant genotypes were produced. This includes those generated by transgenic approaches, gene editing, chemical/radiation-based mutagenesis and hybridization. For transgenic lines, describe the transformation method, the number of independent lines analyzed and the generation upon which experiments were performed. For gene-edited lines, describe the editor used, the endogenous sequence targeted for editing, the targeting guide RNA sequence (if applicable) and how the editor was applied.

Authentication

Describe any authentication procedures for each seed stock used or novel genotype generated. Describe any experiments used to assess the effect of a mutation and, where applicable, how potential secondary effects (e.g. second site T-DNA insertions, mosiacism, off-target gene editing) were examined.

Flow Cytometry

Plots

Confirm that:

☒ The axis labels state the marker and fluorochrome used (e.g. CD4-FITC).

☒ The axis scales are clearly visible. Include numbers along axes only for bottom left plot of group (a 'group' is an analysis of identical markers).

☒ All plots are contour plots with outliers or pseudocolor plots.

☒ A numerical value for number of cells or percentage (with statistics) is provided.

Methodology

Sample preparation

Sample preparation of the individual flow cytometry experiments are described in detail in the methods part of the manuscript.

Instrument

FACS Canto II cytometer, Fortessa HTS, BD Aria II (BD Biosciences)

Software

FlowJo 10.0.8 (BD)

Cell population abundance

Describe the abundance of the relevant cell populations within post-sort fractions, providing details on the purity of the samples and how it was determined.

Gating strategy

Gating strategies are described in the manuscript

☐ Tick this box to confirm that a figure exemplifying the gating strategy is provided in the Supplementary Information.
