# Supplementary material for: Dual Ribosome Profiling reveals metabolic limitations of cancer and stromal cells in the tumor microenvironment
Source: Nat Commun. 2025 May 19;16:4652. doi: 10.1038/s41467-025-59986-7 (PMC12089342; doi:10.1038/s41467-025-59986-7)
Supplement: Supplementary file 4 — Description of Additional Supplementary Files [file 41467_2025_59986_MOESM4_ESM.pdf]

Description of Additional Supplementary Files

Supplementary Data 1

Oligonucleotide sequences used in this study
